# Plasmid-borne colistin resistance gene *mcr-3* in *Salmonella* isolates from human infections, Denmark, 2009–17

**DOI:** 10.2807/1560-7917.ES.2017.22.31.30587

**Published:** 2017-08-03

**Authors:** Eva Litrup, Kristoffer Kiil, Anette M Hammerum, Louise Roer, Eva M Nielsen, Mia Torpdahl

**Affiliations:** 1 Department of Bacteria, Parasites and Fungi, Statens Serum Institut, Copenhagen, Denmark

**Keywords:** mcr-3, colistin resistance, Salmonella, plasmid, ESBL, antimicrobial resistance

## Abstract

This report describes one Salmonella isolate harbouring both *mcr-1* and *mcr-3*. We also found nine other Salmonella isolates positive for the plasmid-borne colistin resistance gene, *mcr-3*. The strains were isolated from patients in Denmark between 2009 and 2017 and five of the patients had travelled to Asia. In addition to *mcr-3*, all strains were found positive for *bla_TEM-1_*, *strA*, *strB*, *sul2* and *tet*(A) or *tet*(B), and most strains were positive for *bla_CTX-M-55_* and *qnrS*.

In June 2017, Yin et al. reported a third plasmid-borne colistin resistance gene (encoded by *mcr-3*) in *Escherichia coli* from China [1]. The same study identified a truncated transposon element immediately upstream of *mcr-3* that was also found in *E. coli*, *Klebsiella pneumoniae* and *Salmonella* Typhimurium [1]. This followed reports from 2016 on the plasmid-borne colistin resistance genes *mcr-1* [2] and *mcr-2* [3] and the subsequent reporting of global spread of *mcr-1* and detection of *mcr-1* from livestock/retail meat and human sources [4,5]. The worldwide spread of plasmid-mediated colistin resistance is of major concern, as colistin in some instances is the drug of last resort for multidrug-resistant infections.

## Detection of *mcr-3*-positive human clinical isolates in Denmark

Statens Serum Institut (SSI) receives isolates (originating from stool, blood or urine samples) and patient information from all human clinical *Salmonella* infections for surveillance in Denmark. Since January 2017, all *Salmonella* isolates have been subjected to whole genome sequencing (WGS) in real time, in contrast to previous years where only a selection of isolates were sequenced. In a retrospective search for the truncated transposon and *mcr-3* [1] among all Danish *Salmonella* isolates sequenced at SSI between 2009 and 2017 (ca 2,500), 10 *Salmonella* isolates were found positive for the *mcr-3* gene; all isolates were ST34 and of serovar Typhimurium or monophasic variants of Typhimurium (4,5,12:i:- and 4,12:i:-) (Table). The years of disease onset recorded for the 10 Salmonella isolates were 2009 (n = 1), 2010 (n = 1), 2011 (n = 2), 2012 (n = 3), 2015 (n = 1), 2016 (n = 1) and 2017 (n = 1). Four of the patients had travelled to Thailand and one to Vietnam before onset of disease, three patients had unknown travel history and two patients had acquired their infection domestically (Table).

**Table:** *Salmonella* isolates positive for *mcr-3*, Denmark, 2009–17 (n=10)

Isolate	Year of isolation	Serotype	Travel reported	Inc type	Resistance genes detected by ResFinder besides *mcr-3*
SSI-AC256	2009	Typhimurium	None	IncA/C2, ColRNAI	*aac(3)-IId, bla_CTX-M-55_, bla_TEM-1B_, catA2, floR, qnrS1, strA, strB, sul2, tet*(A)
SSI-AC257	2010	O:4,5,12; H:i:-	Unknown	IncA/C2, ColRNAI	*aac(3)-IId, bla_CTX-M-55_, bla_TEM-1B_, floR, qnrS1, strA, strB, sul2, tet*(A)
SSI-AC259	2011	O:4,5,12; H:i:-	Thailand	IncFII, IncQ1	*aac(3)-IId, bla_CTX-M-55_, bla_TEM-1B_, catA2, qnrS1, strA, strB, sul2, tet*(B)
SSI-AC258	2011	O:4,5,12; H:i:-	Thailand	IncA/C2	*aac(3)-IId, bla_CTX-M-55_, bla_TEM-1B_, catA2, floR, qnrS1, strA, strB, sul2, tet*(A)
SSI_AA940	2012	Typhimurium	None	ColRNAI	*aac(3)-IId, bla_CTX-M-55_, catA2, floR, qnrS1, strA, strB, sul2, tet(A)*
SSI_AA992	2012	O:4,12; H:i: -	Unknown	IncHI2A, IncHI2, IncN, TrfA, IncQ1, ColRNAI	*aadA2, aph(3')-Ic, bla_TEM-1B_, dfrA12, strA, strB, sul1, sul2, tet(A), tet(B)*
SSI_AA934	2012	Typhimurium	Thailand	IncA/C2, ColRNAI	*aac(3)-IId, bla_CTX-M-55_, bla_TEM-1B_, catA2, floR, qnrS1, strA, strB, sul2, tet*(A)
SSI-AC260	2015	O:4,12; H:i: -	Vietnam	IncA/C2	*bla_CTX-M-55_, bla_TEM-1B_, floR, strA, strB, sul2, tet*(A)
SSI-AC261	2016	O:4,5,12; H:i:-	Thailand	IncFII(pCoo), IncX1, Col(MP18), IncQ1,ColRNAI	*bla_TEM-1B_, strA, strB, sul2, tet*(B)
SSI-AC262	2017	O:4,12; H:i: -	Unknown	IncFIC(FII), IncI2, IncA/C2	*bla_CTX-M-55_, bla_TEM-1B_, floR, mcr-1, strA, strB, sul2, tet*(A)

## Single-nucleotide polymorphism-based phylogeny

From WGS data (Illumina MiSeq) of the 10 *mcr-3* positive isolates, extraction of sequences types (http://enterobase.warwick.ac.uk) assigned all isolates to ST34 (Table). The sequence data was used to create a single-nucleotide polymorphism (SNP) phylogeny using the Northern Arizona SNP Pipeline (NASP) [6], including local sequences representing the diversity within ST34 isolated from patients in Denmark with different travel histories (Figure). In total, 361 *Salmonella* isolates with serotypes Typhimurium and the monophasic variants of Typhimurium were included in the SNP analysis and the 10 *mcr-3*-positive isolates were located in two clades of the phylogeny (Figure). These two clades also contained most of the *Salmonella* isolates from patients with known travel history to Asia. Isolates from patients that had travelled in Europe, were domestically acquired or of unknown origin, were evenly distributed in the tree.

**Figure:**
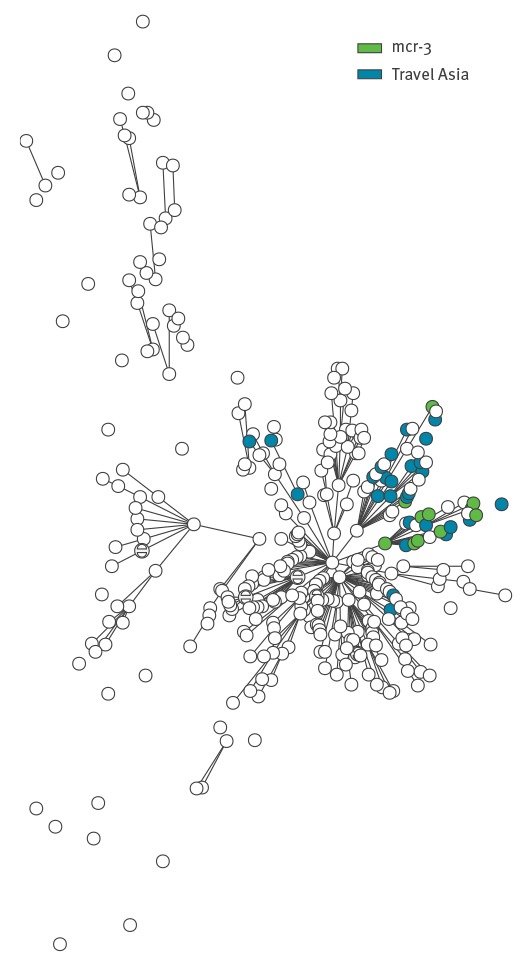
Phylogenetic tree calculated on core SNPs, including 361 isolates and 2,925 SNPs

SNP: single-nucleotide polymorphism.

Green nodes: *mcr-3*-positive isolates; blue nodes: isolates with confirmed patient travel to Asia.

## Characterisation of isolates

Using ResFinder (https://cge.cbs.dtu.dk/services/ResFinder/), various resistance genes were detected in the WGS data (Table). The most prevalent resistance genes found in relation to the monophasic variant of Typhimurium, *bla*_TEM − 1_, *strA-strB, sul2* and *tet*(A) or *tet*(B) [7] were detected in all *mcr-3*-positive isolates in this study. In addition, eight of the isolates carried *bla*_CTX-M-55_ and six isolates contained *qnrS1,* conferring low level resistance to fluoroquinolones [8]. Of note, one isolate from 2017 was positive to both *mcr-1* and *mcr-3* [2]. Using PlasmidFinder (https://cge.cbs.dtu.dk/services/PlasmidFinder/), several different replicons were detected (Table). One was IncHI2, the same as the plasmid-borne *mcr-3* identified by Yin et al. on a 261-kb IncHI2-type plasmid (pWJ1) [1].

## Discussion

The several recent publications that describe new plasmid-mediated colistin resistance [1-3] are of great concern and underline the importance of monitoring the spread of these plasmids worldwide [4,5]. In Denmark, *mcr-1*-positive isolates from human cases with *Salmonella* infection have been reported [9], whereas *mcr-2* was not found among Danish isolates. This report describes 10 *mcr-3*-positive isolates from human *Salmonella* infections between 2009 and 2017. One of the isolates is especially relevant as it was positive for both *mcr-1* and *mcr-3* and to our knowledge a rare combination of two colistin resistance genes. 

Eight of the MCR-3-producing *Salmonella* isolates from the Danish patients were also positive for *bla*_CTX-M-55_. CTX-M-55 was first reported from Thailand [10], and the *bla*_CTX-M-55_ gene is a prevalent gene in Gram-negative bacteria isolated from animals and humans in Asia [11-13]. Furthermore, *bla*_CTX-M-55_-positive monophasic *Salmonella* Typhimurium has been isolated from patients who reported travel to Thailand [14]. Several other studies have detected isolates with both *mcr-1* and *bla*_CTX-M-55_, most of them also with a link to Asia [4,15]. Half of the Danish *mcr-3*-positive cases were linked with travel to Asia within one week before onset of disease. In general, the level of resistance in the Danish human cases of *Salmonella* Typhimurium and the monophasic variant is higher in cases with travel history than in domestically acquired cases [16]. 

One of the major concerns with the introduction of plasmid-borne colistin resistance is its ability of rapid horizontal spread between and within bacterial species; therefore plasmid spread could result in an increase in colistin-resistant bacteria. This in combination with the detection of MCR-3-producing isolates in patients without travel history is worrying because *mcr-3* could in the future be present in food-borne outbreaks with *Salmonella* or *E. coli.*
